# In the presence of *Trypanosoma cruzi* antigens, activated peripheral T lymphocytes retained in the liver induce a proinflammatory phenotypic and functional shift in intrahepatic T lymphocyte

**DOI:** 10.1002/JLB.3A0220-399RR

**Published:** 2020-03-23

**Authors:** Marcelo Meuser‐Batista, Natalia Vacani‐Martins, Cynthia Machado Cascabulho, Daniela Gois Beghini, Andrea Henriques‐Pons

**Affiliations:** ^1^ Laboratório de Inovações em Terapias Ensino e Bioprodutos from Instituto Oswaldo Cruz Rio de Janeiro Brazil; ^2^ Depto de Anatomia Patológica e Citopatologia Instituto Fernandes Figueira. Fundação Oswaldo Cruz Rio de Janeiro Brazil

**Keywords:** hepatic T lymphocytes, liver immune response, peripheral T cells, hepatic immunoregulation, pathogen‐associated molecular pattern (PAMP), *Trypanosoma. cruzi*

## Abstract

In secondary lymphoid organs, pathogen‐derived and endogenous danger molecules are recognized by pattern recognition receptors, leading to adaptive proinflammatory immune responses. This conceptual rule does not apply directly to the liver, as hepatic immune cells tolerate gut‐derived bacterial molecules from the flora. Therefore, the recognition of danger and proinflammatory stimuli differs between the periphery and the liver. However, the tolerant nature of the liver must be overcome in the case of infections or cancer, for example. The central paradigm is the basis for danger recognition and the balance between inflammation and tolerance in the liver. Here, we observed functional integration, with activated peripheral T lymphocytes playing a role in the induction of a proinflammatory environment in the liver in the presence of *Trypanosoma cruzi* antigens. When only parasite extract was orally administered, it led to the up‐regulation of hepatic tolerance markers, but oral treatment plus adoptively transferred activated splenic T lymphocytes led to a proinflammatory response. Moreover, treated/recipient mice showed increased levels of TNF, IFN‐γ, IL‐6, and CCL2 in the liver and increased numbers of effector and/or effector memory T lymphocytes and F4/80^+^ cells. There was a reduction in FoxP3^+^ Treg cells, NKT cells, and γδ T lymphocytes with increased liver damage in the presence of activated peripheral T cells. Our results show that the induction of a proinflammatory liver response against *T. cruzi* danger molecules is at least partially dependent on cooperation with activated peripheral T cells.

## Graphical abstract

Activated peripheral T lymphocytes exert direct and indirect functions in the liver, leading to hepatic T lymphocytes expansion and activation in the presence of *T. cruzi* antigens.

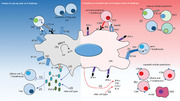

**Abbreviations**
AIH: autoimmune hepatitis
DAMPs: damage‐associated molecular patterns
IHLs: intrahepatic lymphocytes
KCs: Kupffer cells
LSECs: liver sinusoidal endothelial cells
PAMPs: pathogen‐associated molecular patterns
PRRs: pattern recognition receptors
Treg: regulatory T cells

## 1 INTRODUCTION

The immune response in mammals is composed of an intricate network of positive and negative signals that ultimately determine whether or not a potentially antigenic molecule will trigger an inflammatory response. For peripheral immune responses, the antigens must be recognized as danger signals, which are either endogenous damage‐associated molecular patterns (DAMPs)^1^ or exogenous pathogen‐associated molecular patterns (PAMPs).^2^ These molecules are then recognized by membrane, endosomal, or cytoplasmic pattern recognition receptors (PRRs)^3^, ^4^ expressed on APCs, which induce the proinflammatory T cell response. Tolerance mechanisms play a central role in this process by dampening T lymphocyte activation in response to harmless or self‐molecules and rely on cells such as tolerogenic dendritic cells and regulatory T (Treg) cells,^5^ for example.

The induction of effector immune responses in the gut and liver are controlled by a nonclassical set of conceptual pathways. In these organs, APCs are constantly exposed to high levels of food proteins and PAMPs from bacterial components of the flora, such as LPS. An environment that would be sensed as rich in proinflammatory danger signals in the periphery, leads to immunologic tolerance in the liver and intestines. In the liver, this tolerance must be overcome in the case of hepatic cancer and viral infections, for example. The central paradigm is that the perception of danger differs enormously between the periphery and the liver, with similar PAMPs being sensed as classic proinflammatory molecules in the periphery and tolerogenic stimuli in the liver.

Our knowledge about liver immune cells and function has significantly improved in recent years. We know several infectious or tumorous conditions, biochemical pathways, and cellular interactions that compose a resultant proinflammatory response in the liver. However, a primary question remains open, and we do not know how the liver discriminates which DAMPs/PAMPs should be recognized as danger molecules for the initiation of an effector immune response. As the liver harbors stromal and sinusoidal hepatic cells that remain constantly exposed to several intestinal PAMPs, the trigger to overcoming tolerance may not reside in the liver itself.

In this work, we used experimental *Trypanosoma cruzi* infection and parasite antigens to study hepatic tolerance and inflammation. After the infection, Kupffer cells (KCs) and hepatocytes are infected by the parasite,^6^ there is hepatomegaly,^7^ hepatocyte hypertrophy,^8^ and an enrichment of NK cells and CD4, CD8, and γδ T lymphocytes producing IFN‐γ in the liver.^9^ Taken together, our data showed the relevance of danger molecules recognition in peripheral secondary lymphoid sites and activated T cells in altering the phenotype and function of intrahepatic T lymphocytes, changing the balance of hepatic tolerance towards a local proinflammatory response.

Other results support a correlation that is not yet clearly established between peripheral T cells and the hepatic inflammatory response. To date, Bowen elegantly showed that when T lymphocytes are prevented from entering lymph nodes after antibody‐induced CD62‐L neutralization, liver damage was no longer observed in transgenic mice with autoimmune hepatitis (AIH).^10^ However, the authors proposed that there was a competition for primary activation of CD8^+^ T cells between secondary lymphoid tissues and the liver and that the initial site of activation would determine the resultant inflammatory response or tolerance in the liver. In this case, if the antigens were primarily recognized in the liver, immune tolerance would be the defined response. It was also proposed that antigen‐primed T cells would migrate from the lymph nodes and be retained in the liver for activation when in the presence of a more proinflammatory environment, as in the case of secondary antigen exposure.^11^ Zeng et al.^12^ showed that an IL‐12‐based vaccination therapy reversed immune tolerance in the liver, and other authors investigated the importance of the site of antigen expression in hepatic tolerance.^13^, ^14^

However, in these experimental approaches, the expression of antigens is not restricted to the hepatic environment or the periphery, and this mixed antigen site presentation impedes the evaluation of the primary site of danger recognition. Our results showed that adoptively transferred peripheral T lymphocytes that were activated by *T. cruzi* antigens were retained in the liver and led to hepatic T cell activation, which in turn partially subverted the tolerant nature of the liver. This peripheral‐dependent induction of a more proinflammatory status of lymphoid and myeloid cells in the liver may provide new ways to understand hepatic pathologies.

## 2 MATERIALS AND METHODS

### 2.1 Mice

Male, pathogen‐free C57BL/10 and C57BL/6 mice were obtained from the FIOCRUZ animal facility (ICTB) or the Biotério Central of Universidade de Minas Gerais. C57Bl/6 GFP mice were kindly provided by Dr. Regina Coeli Goldenberg and Dr. Isalira Ramos from the Instituto de Biofísica Carlos Chagas Filho at the Universidade Federal do Rio de Janeiro. All mice were housed for at least 1 week before experimentation under conditions complying with the “Guide for the Care and Use of Laboratory Animals” (DHEW Publication No. [NIH] 80‐23, 1996). The FIOCRUZ Committee of Ethics in Research approved this project (L006/15), according to resolution 196/96 of the National Health Council of the Brazilian Ministry of Health.

### 2.2 Liver cell isolation

The livers were extensively perfused through the portal vein using DMEM (Thermo Fisher Scientific, Waltham, Massachusetts, USA), part of the right medial lobe was fragmented, and the cells were obtained by maceration. All samples were filtered using a 40 µm strainer (Greiner Bio‐one, Frickenhausen, Germany) and washed in DMEM medium containing 10% FBS (Thermo Fisher). For flow cytometry, the samples were incubated for 30 min with 10% inactivated sheep serum in DMEM to block Fcγ receptors. All cells were maintained on ice until use.

### 2.3 Splenic cell isolation

The spleens were collected and macerated, and the cells were washed twice in DMEM medium containing 10% FBS. Pelleted cells were resuspended in 1 mL of red blood cell lysis solution (BioLegend, San Diego, CA, USA) and then incubated in 1 mL of the FcγR blocking solution.

### 2.4 *T. cruzi* infection

For experimental infection, bloodstream trypomastigote forms of the *T. cruzi* Y strain were obtained from infected Swiss‐Webster mice after 7 days of infection. The parasites were counted, and the inocula were adjusted in PBS as indicated in the figure legends. The animals were divided into the following groups: “control,” which received 1 i.p. injection of PBS; “i.p.,” which received i.p. infection using 1 × 10^4^ trypomastigote forms of *T. cruzi*; “oral,” which received oral infection using 1 × 10^7^ parasites in the oral cavity; and “OralAgTc,” which received *T. cruzi* antigen (extract) administered by gavage at a dose equivalent to 1 × 10^7^ parasites per mouse. The *T. cruzi* extract was produced by heating the samples at 80°C for 20 min, followed by 3 cycles of freezing and thawing. Blood parasitemia was counted in 5 microliters of blood collected from tail snips.

### 2.5 Flow cytometry

For flow cytometry analysis, isolated cells from the spleen or liver were counted and distributed in 96‐well round‐bottom plates (3 × 10^5^ hepatic and 1 × 10^6^ splenic cells per well) for monoclonal antibody labeling. All antibodies were previously titrated and purchased from BioLegend. Cell viability was evaluated by 7AAD (BioLegend) or the Live/Dead kit (Thermo Fisher) according to the manufacturer's instructions. The samples were acquired using a Cyan ADP flow cytometer (Beckman Coulter, Brea, CA, USA) at the Multiparametric Multiuser Flow Cytometry Facility at the Instituto Oswaldo Cruz, and the data were analyzed using Summit (version 6.1) or FlowJo software. Doublet exclusion was performed in FSC‐H x FSC‐A dot plots.

### 2.6 Adoptive transfer of activated splenic T lymphocytes

GFP or wild‐type (WT) donor mice, as indicated in the figure legends, received 1 weekly i.p. injection of *T. cruzi* extract (equivalent to 1 × 10^7^ parasites per mouse) in 6 mg of aluminum hydroxide (Sigma–Aldrich, St Louis, MO, USA) for 2 weeks. Seven days after the second treatment, activated splenic T lymphocytes (CD3^+^CD44^high^CD62‐L^−^CD197^−^) were purified by flow cytometry using a MoFlo Astrius flow cytometer (Beckman Coulter) at the Multiuser Cell Sorting facility at the Instituto Oswaldo Cruz. Only samples with at least 95% purity and viability were used. Experimental groups were then divided according to the oral treatment using parasite extract (equivalent to 1 × 10^7^ parasites) and/or i.p. cellular transfer of purified, activated splenic T cells (5 × 10^4^ cells/mouse), as shown in Table 1.

**TABLE 1:** Experimental groups for adoptive cellular transfer

Group	Oral treatment		i.p. treatment
Control	PBS	and	PBS
T cell	PBS	and	T cell transfer
T cell+AgTc	Parasite extract	and	T cell transfer
AgTc	Parasite extract	and	PBS

Oral and i.p. treatments were performed at the same time and were a single dose. Fifteen days after the treatments, all mice received *T. cruzi* extract by gavage (equivalent to 1 × 10^7^ parasites) and were euthanized after 36 h, as indicated in the figure legends.

### 2.7 Adoptive transfer of activated hepatic T lymphocytes

Activated splenic T lymphocytes were purified from *T. cruzi* extract‐treated GFP mice, as detailed in item 6. These cells were i.p. transferred to C57BL/6 WT mice that concomitantly received parasite extract by gavage (equivalent to 1 × 10^7^ parasites). Fifteen days after the adoptive cell transfer, activated hepatic GFP^−^ T lymphocytes (CD3^+^CD44^high^CD62‐L^−^CD197^−^) were purified by flow cytometry, and 1 × 10^6^ cells were transferred to new recipient mice that were immediately infected with 1 × 10^4^ trypomastigote forms of the *T. cruzi* Y strain. Fifteen days after this cell transfer and infection, all mice were euthanized, and the samples were collected for analysis.

### 2.8 Immunohistochemistry and H&E staining

Liver fragments were collected before hepatic perfusion, frozen using cold isopentane, and kept in liquid nitrogen until use. FcγRs were blocked using the same solution used for flow cytometry labeling, and sample slices were incubated overnight with anti‐B7‐H1, F4/80, and IDO‐1 primary antibodies (all from BioLegend) at room temperature with agitation. The slices were then incubated with an Immpress peroxidase anti‐rat polymer detection kit (Vector Laboratories, Burlingame, CA, USA), and the visualization was performed using a DAB peroxidase (HRP) substrate kit (with nickel), 3,3′‐diaminobenzidine and nickel (Vector Laboratories). Quantification of positive labeling was determined by scanning the whole tissue in approximately 30 individual microscopic fields per sample using ImageJ software (NIH Image, Bethesda, MA, USA). Frozen sections of nonperfused livers were also stained using Harris hematoxylin & eosin.

### 2.9 Cytokine measurement

Plasma samples from heparinized blood, liver extracts from perfused livers, and spleen extracts were used for the detection of cytokines and chemokines by flow cytometry, as recommended by the manufacturers. For tissue analysis, sample fragments were incubated in ice‐cold extraction buffer (1% NP‐40, leupeptin 1 mmol/L, phenylmethylsulfonyl fluoride 100 mmol/L, pepstatin A 1 mmol/L, and EDTA 100 mmol/L; all purchased from Sigma–Aldrich) and centrifuged (500×*g*), and the supernatants were frozen until use. We used the CBA inflammation and CBA Th1/Th2/Th17 kits, both from BD Biosciences (San Jose, CA, USA), and the chemokine FlowCytomix multiplex kit (eBioscience, San Diego, CA, USA). The samples were acquired using a FACSCalibur flow cytometer (BD Biosciences, CA, USA), and data analysis was performed using the CBA analysis FCAP software (BD Biosciences) or the FlowCytomix software (eBioscience) for chemokines. All results are expressed as pg of cytokine per mL in plasma or per mg of total proteins per sample in tissues. Protein concentration was individually determined using a BCA kit (Thermo Fisher). TGF‐β was evaluated using a Legend Max free active TGF‐β ELISA kit with precoated plates according to the manufacturer's instructions (BioLegend).

### 2.10 Dot blotting

After liver perfusion, total proteins were extracted from the tissue fragments for the detection of CTLA‐4 and PD‐1 by dot blotting, as previously described.^15^

### 2.11 Biochemistry (ALT, AST, CK‐NAC, and CK‐MB)

Creatine kinase (CK) cardiac isoform (CK‐MB) and total CK (CK‐NAC) were used to evaluate muscle damage, the transaminases ALT and AST were used to evaluate liver damage, and all were evaluated in the plasma. Blood samples were collected from tail snips in heparinized capillary tubes, centrifuged, and analyzed using commercially available kits (all from Wiener Lab, Rosario, Argentine). The CK results are expressed as the rate of NADPH increase (ΔE/min) in 3 sequential readings by a spectrophotometer (Molecular Devices, San Jose, CA, USA) at 340 nm.

### 2.12 Statistical analysis

All data were expressed as arithmetic mean ± SD. Before the statistical analysis, we used the Shapiro–Wilk test to verify if the samples had a normal distribution, then we used 1‐way ANOVA followed by Tukey's posttest for normal samples. When the data did not follow a Gaussian distribution, we used Kruskal–Wallis nonparametric test followed by Dunn's test. The adopted significance level was *α* = 5% (*P* < 0.05) and we used R (R Core Team, 2019). R: A language and environment for statistical computing. R Foundation for Statistical Computing, Vienna, Austria. URL https://www.R-project.org/.) for all statistical analysis.

## 3 RESULTS

### 3.1 The parasite antigen entry route determines the immune responses elicited in the liver

**FIGURE 1:**
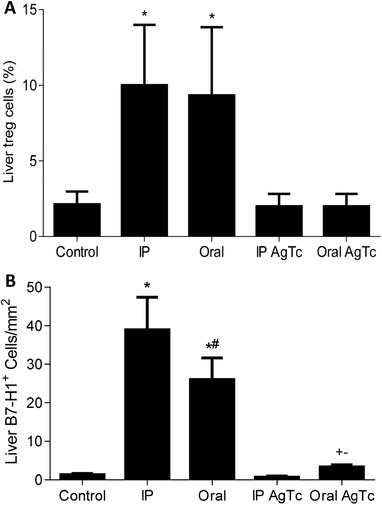
**Evaluation of Treg cells and B7‐H1 expression in the liver**. Thirteen‐week‐old male C57BL/10 mice were grouped as Control, infected (oral or i.p.), and mice that received *T. cruzi* extract i.p. (IPAgTc) or by gavage (OralAgTc). After 15 days, liver Treg cells (CD3^+^CD4^+^FoxP3^+^) were evaluated by flow cytometry (**A**), and the percentage of B7‐H1^+^ area (**B**) in hepatic tissue was assessed by immunohistochemistry. Data represent the mean and standard deviation of 5 independent experiments with 5–6 animals per group. The results were analyzed using ANOVA and Tukey's as the post hoc test and * indicates *P* < 0.05 compared with the Control, IPAgTc and OralAgTc groups; # indicates *P* < 0.05 compared with the i.p. group; + indicates *P* < 0.05 compared with the Control group; and ^−^ indicates *P* < 0.05 compared with the IPAgTc group. Bar: 50 µm

Approximately 80% of the liver blood supply arrives through the hepatic portal vein, with blood rich in gut‐derived microbiota molecules, and it has been proposed that the constant exposure of liver cells to commensal PAMPs leads to the observed tolerance in this organ. We therefore decided to treat groups of mice with *T. cruzi* extract by gavage, as they had not been previously exposed to these PAMPs, and evaluate whether the hepatic environment would tolerate these molecules. We observed no increase in FoxP3^+^ Treg cells after i.p. or oral administration of parasite extract (Fig. 1A). Moreover, there was a discrete but significant increase in B7‐H1^+^ area only in the OralAgTc group (Fig. 1B), which was apparently restricted to liver sinusoidal endothelial cells (LSECs) and inflammatory cells (data not shown). After i.p. or oral infection, there was an increase in both FoxP3^+^ cells and B7‐H1^+^ area (Fig. 1), a molecule that binds to PD1 and is associated with T lymphocyte down‐regulation. The analysis of cytokines in the hepatic stroma showed that when parasite extract was given by gavage (OralAgTc group), there was an increase in the anti‐inflammatory cytokine IL‐10, but no increase in TGF‐β (Supplemental Fig. 1), when compared with those of the Control group. There was also no increase in the cytokines TNF, IFN‐γ, RANTES, IL‐6, and MIP‐1α (Supplemental Fig. 1), all of which are associated with proinflammatory functions. On the other hand, other proinflammatory cytokines increased in the OralAgTc group, such as MCP‐3, a chemokine that attracts monocytes and can regulate macrophage function, and IL‐17A (Supplemental Fig. 1). These results illustrate the hepatic balance between inflammatory and tolerant pathways. However, nonportal delivery of antigens (IPAgTc group) led to an increase in only IL‐10, TNF, and IL‐6 (Supplemental Fig. 1). In the livers of infected mice (i.p. and oral groups), there was also an increase in anti and proinflammatory cytokines, including IL‐10, TGF‐β, TNF, IFN‐γ, RANTES, IL‐6, MCP‐3, and MIP‐1α (only in the i.p. group), when compared with those of the Control group (Supplemental Fig. 1). This result was different from that of the spleen, where only the proinflammatory cytokines IFN‐γ, TNF, and IL‐6 increased after infection, with no significant differences in the levels of IL‐2, IL‐17A, and IL‐10 when comparing the levels in all groups (data not shown). We also measured IL‐2, MIP‐1β, and GM‐CSF in all liver samples, but there were no differences in these cytokines in any of the experimental groups (data not shown).

At this point, our data suggested that the liver responds differently to the same pool of *T. cruzi* PAMPs that reaches the hepatic stroma primarily through the hepatic portal vein (OralAgTc group) or the hepatic artery (IPAgTc group) (systemic pathway). However, this possibility has not been conclusively demonstrated, as it is likely that the kinetics, total amount, and nature of the PAMPs that arrive in the liver are quite different with these 2 discrete routes of administration (oral vs. i.p.). Despite experimental limitations, this first part of the results helped us to formulate our main hypothesis, which is that the recognition of danger molecules by T lymphocytes in the lymph nodes leads to retention of activated peripheral T cells in the liver and then to the shifting of intrahepatic lymphocytes (IHLs) towards a proinflammatory phenotype and response.

### 3.2 Adoptive transfer of activated peripheral T lymphocytes leads to a proinflammatory balance in the liver

**FIGURE 2:**
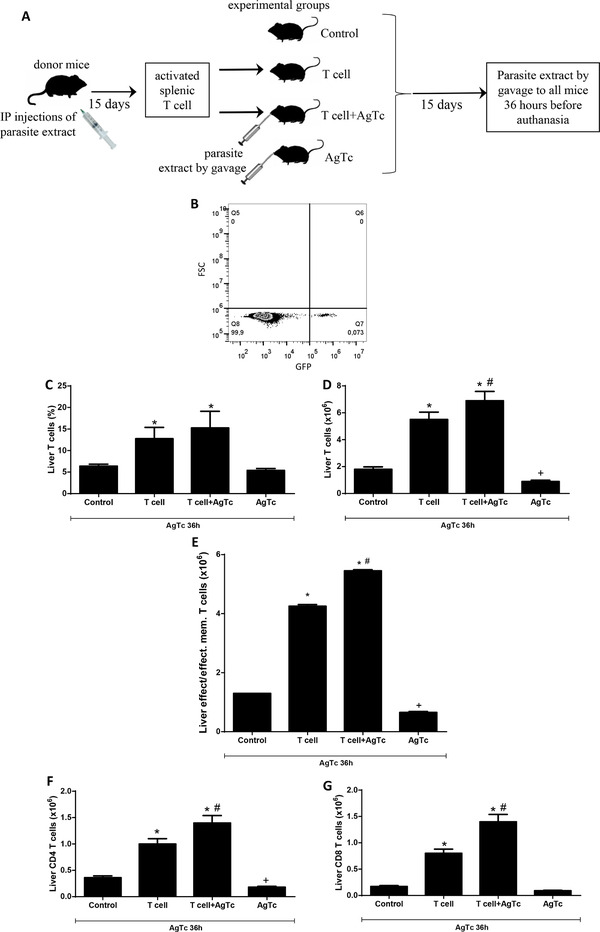
**Adoptive transfer of activated peripheral T lymphocyte and intrahepatic T lymphocyte subpopulations**. Thirteen‐week‐old male C57BL/10 donor mice received 2 i.p. injections of *T. cruzi* extract, and activated splenic T lymphocytes (CD3^+^CD44^high^CD62‐L^−^CD197^−^) were purified after fifteen days (**A**). Then, 5–7 syngeneic mice were divided per group between the following groups: control; T cell; T cell+AgTc; and AgTc. Fifteen days after T cell transfer and/or antigen treatment, all mice received parasite extract (equivalent to 1 × 10^7^ parasites) by gavage and after additional 36 h they were euthanized (**A**). To confirm that donor T lymphocyte were retained in the liver stroma for resident IHLs activation, independent experiments were done using 13‐week‐old male GFP mice (C57BL/6 background) that were treated twice with parasite extract and the analysis of transferred GFP^+^ T cells in the C57BL/6 recipient liver stroma was performed in the morphologic lymphocyte gate to identify donor GFP^+^ cells (**B**). The percentage (**C**) and number (**D**) of intrahepatic T lymphocyte were analyzed in the morphologic lymphocyte gate of recipient mice. The percentage of effector/effector memory T (T_em_) cells (CD3^+^CD62‐L^−^CD127^+/−^CD44^high^CD197^−^) was analyzed in the morphologic lymphocyte gate and CD3^+^ cells (**E**), as well as the number of CD4^+^ (**F**) and CD8^+^ (**G**) T cells. Data represent the mean and standard deviation of 3 independent experiments with 5 animals per group. The results were analyzed using ANOVA and Tukey's as the post hoc test and * indicates *P* < 0.05 compared with the Control and AgTc groups; # indicates *P* < 0.05 compared with the T cell group; + indicates *P* < 0.05 compared with the Control group

To test the possible relevance of the periphery on the hepatic response to danger and inflammation in this infectious model, we i.p. treated donor mice with *T. cruzi* extract and purified activated peripheral (splenic) T lymphocytes. These cells were then transferred to syngeneic recipient mice that did or did not receive parasite extract by gavage (Fig. 2A). Two weeks after the cell transfer and/or treatment, all mice received parasite extract by gavage and were euthanized after 36 h. We confirmed that adoptively transferred activated T lymphocytes are retained in the liver by using GFP mice as the T cell donors in an independent experiment (Fig. 2B), and GFP^+^ CD3^+^ T cells represented 0.07–0.1% of the events (Fig. 2B) in the lymphocyte gate of recipient mice. We observed that the adoptive transfer of only 5 × 10^4^ activated peripheral T lymphocytes modulated the population of hepatic T lymphocytes, with increased relative (Fig. 2C) and absolute numbers (Fig. 2D) of TCRαβ^+^NK1.1^−^CD3^high^ IHLs only in the groups that received T cells (T cell and T cell+AgTc) (analysis of the lymphocytes gate).

The effect of activated peripheral T cell transfer on the phenotype of hepatic T lymphocytes was evaluated in the lymphocyte and CD3^+^ gates for effector and T_em_ cells (CD44^high^CD62L^−^CD127^+/−^CD197^−^) (Fig. 2E). In this analysis, there was an increase in the number of effector/T_em_ cells in the T cell and T cell+AgTc groups compared with those of the control mice and mice that received only oral parasite extract (AgTc group) (Fig. 2E). After the adoptive transfer of activated peripheral T cells, we found an increase of at least 2.5‐fold in hepatic CD4^+^ T cells in recipient mice (Fig. 2F) and of 4.5‐fold in CD8^+^ T cells (Fig. 2G) in the T cell and T cell+AgTc groups compared with those of the respective controls. These results suggest that activated peripheral T lymphocytes are retained in the liver and lead to an increased number of recipient effector and/or T_em_ lymphocytes in the organ.

**FIGURE 3:**
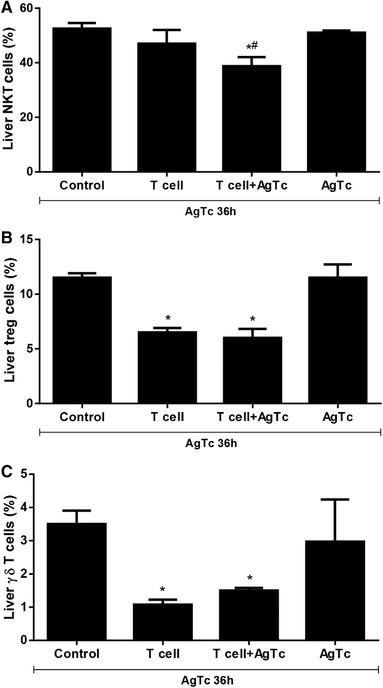
**Flow cytometry evaluation of NKT, Treg cells and γδ T lymphocytes in the liver**. Activated splenic T lymphocytes (CD3^+^CD44^high^CD62‐L^−^CD197^−^) were purified from 13‐week‐old male C57BL/10 mice after i.p. treatment with *T. cruzi* extract. Recipient mice were divided as follows: “Control,” mice that received PBS by gavage; “T cell,” mice that received i.p. administration of 5 × 10^4^ T cells/mouse and PBS by gavage; “T cell+AgTc,” mice that received i.p. administration of 5 × 10^4^ T cells/mouse and parasite extract, equivalent to 1 × 10^7^ parasites by gavage; and “AgTc,” mice that received i.p. PBS and parasite extract, equivalent to 1 × 10^7^ parasites by gavage. Fifteen days after the transfer/treatment, all mice received the equivalent of 1 × 10^7^ parasites by gavage (challenge) and were euthanized after 36 h. The analysis was performed in the morphologic lymphocyte gate and CD3^+^ gate for; NKT (NK1.1^+^CD3^+^) (**A**); Treg (CD3^+^CD4^+^FoxP3^+^) (**B**) cells, and γδ T cells (γδ^+^ TCR) (**C**). Data represent the mean and standard deviation of 3 independent experiments with 5 animals per group. The results were analyzed using ANOVA and Tukey's as the post hoc test and * indicates *P* < 0.05 compared with the Control and AgTc groups; # indicates *P* < 0.05 compared with the T cell group

We analyzed NKT (NK1.1^+^CD3^+^) (Fig. 3A), Treg (CD3^+^CD4^+^FoxP3^+^) (Fig. 3B) cells, and γδ T cells (γδ^+^ TCR) (Fig. 3C) in the morphologic and CD3^+^ cell gate. We found no changes in NK cells when comparing the levels in all groups of mice (data not shown). On the other hand, other lymphoid subpopulations associated with hepatic tolerance were reduced after activated peripheral T lymphocyte transfer, such as NKT cells (Fig. 3B, only in the T cell+AgTc group), FoxP3^+^ Treg cells (Fig. 3C), and γδ T lymphocytes (Fig. 3D).

**FIGURE 4:**
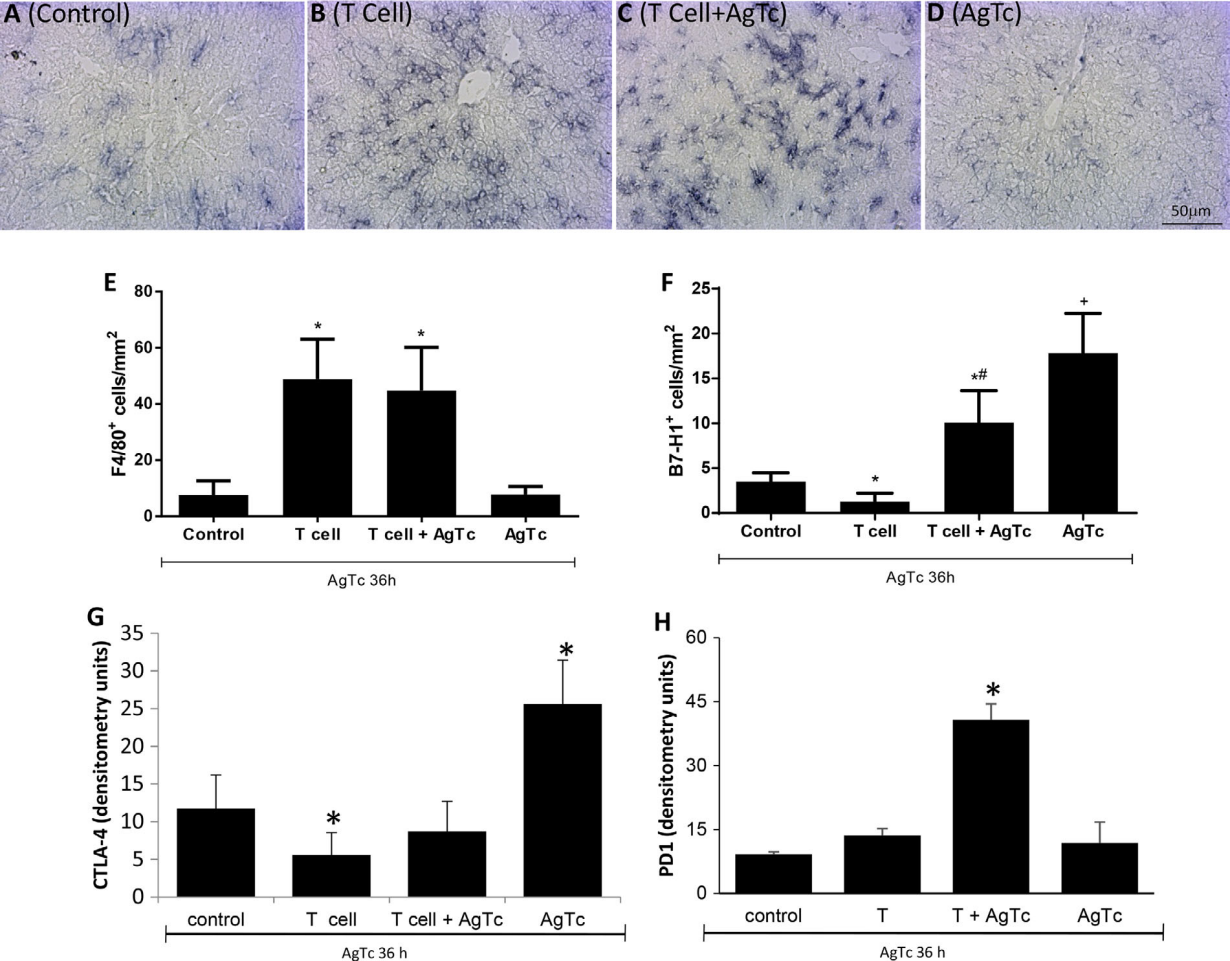
**Evaluation of Kupffer cells and B7‐H1, CTLA‐4, and PD1 expression in the liver after the adoptive transfer of activated peripheral T cells**. Activated splenic T lymphocytes (CD3^+^CD44^high^CD62‐L^−^CD197^−^) were purified from 13‐week‐old male C57BL/10 mice after i.p. treatment with *T. cruzi* extract. Recipient mice were divided as follows: “Control,” mice that received PBS by gavage; “T cell,” mice that received i.p. 5 × 10^4^ T cells/mouse and PBS by gavage; “T cell+AgTc,” mice that received i.p. 5 × 10^4^ T cells/mouse and parasite extract, equivalent to 1 × 10^7^ parasites by gavage; and “AgTc,” mice that received i.p. PBS and parasite extract, equivalent to 1 × 10^7^ parasites by gavage. Fifteen days after the transfer/treatment, all mice received the equivalent of 1 × 10^7^ parasites by gavage and were euthanized after 36 h. F4/80 (**A–E**) and B7‐H1 (**F**) labeling was performed in liver tissue slices, and the quantification of positive cells was performed using ImageJ in 30 microscopic fields per sample. CTLA‐4 (**G**) and PD1 (**H**) were evaluated by dot blotting using a concentration of 80 µg of total liver proteins per dot, and densitometric units were obtained by scanning the membranes. Data represent the mean and standard deviation of 4 independent experiments performed with 5 mice per group. The results were analyzed using ANOVA and Tukey's as post hoc test and * indicates *P* < 0.05 compared with the Control and AgTc groups; # indicates *P* < 0.05 compared with the T cell group; + indicates *P* < 0.05 compared with the Control group. Bar: 50 µm

Nonlymphoid cells in the hepatic environment were also modulated after the adoptive transfer of activated peripheral T lymphocytes, as indicated by the increase in F4/80^+^ cells (Fig. 4A–E) in the T cell and T cell+AgTc groups. Moreover, the expression of B7‐H1 (Fig. 4F) and CTLA‐4 (Fig. 4G), molecules associated with T lymphocyte down‐regulation, was below control levels in the T cell group. On the other hand, both molecules were significantly increased in the AgTc group and expressed at intermediate (B7‐H1) or normal (CTLA‐4) levels in the T cell+AgTc group (Fig. 4). These results suggested that the transfer of T cells restrained the increase in B7‐H1 and CTLA‐4 induced by antigen treatment alone (AgTc group) (Fig. 4F and G) and reinforced the notion that activated peripheral T lymphocytes can control the tolerogenic nature of the liver. However, the expression of PD‐1 increased only in the T cell+AgTc group (Fig. 4H).

**FIGURE 5:**
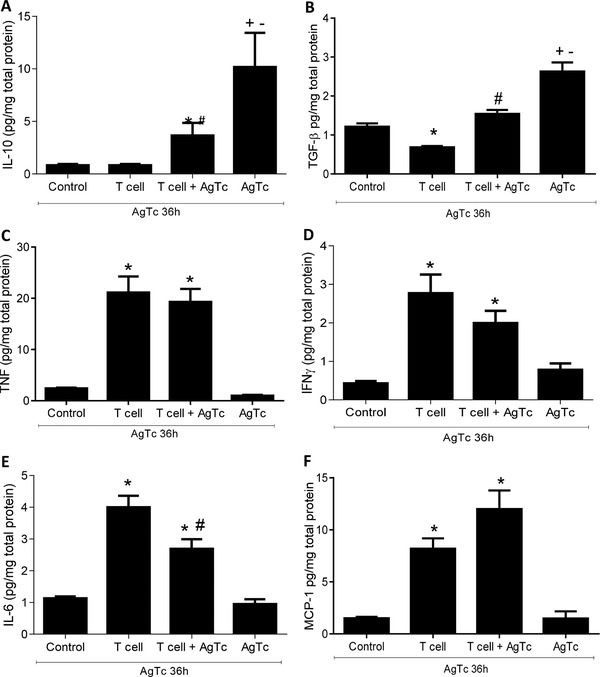
**Evaluation of cytokines in the liver after the adoptive transfer of activated peripheral T cells**. Activated splenic T lymphocytes (CD3^+^CD44^high^CD62‐L^−^CD197^−^) were purified from 13‐week‐old male C57BL/10 mice after i.p. treatment with *T. cruzi* extract. Recipient mice were divided as follows: “Control,” mice that received PBS by gavage; “T cell,” mice that received i.p. 5 × 10^4^ T cells/mouse and PBS by gavage; “T cell+AgTc,” mice that received i.p. 5 × 10^4^ T cells/mouse and parasite extract, equivalent to 1 × 10^7^ parasites by gavage; and “AgTc,” mice that received i.p. PBS and parasite extract, equivalent to 1 × 10^7^ parasites by gavage. Fifteen days after the transfer/treatment, all mice received the equivalent of 1 × 10^7^ parasites by gavage and were euthanized after 36 h. The livers were perfused, and total proteins were extracted for the evaluation of stromal IL‐10 (**A**), TNF (**C**), IFN‐γ (**D**), IL‐6 (**E**), and MCP‐1 (**F**) by flow cytometry. The expression of TGF‐β (**B**) was evaluated by ELISA. The concentration of cytokines was normalized and is expressed in pg per mg of total proteins in tissue extract. Data represent the mean and standard deviation of 3 independent experiments with 5 animals per group. The results were analyzed using Kruskal–Wallis and Dunn's as the post hoc test and * indicates *P* < 0.05 compared with the control and AgTc groups; # indicates *P* < 0.05 compared with the T cell group; + indicates *P* < 0.05 compared with the Control group; ^−^ indicates *P* < 0.05 compared with the T cell and T cell+AgTc groups

When we analyzed hepatic stromal cytokines (Fig. 5A–F), we observed an increase in the proinflammatory cytokines TNF, IFN‐γ, IL‐6, and MCP‐1 in both groups that received activated peripheral T cells. In the AgTc group, only the anti‐inflammatory cytokines IL‐10 and TGF‐β were increased (Fig. 5A–F). It seems, once more, that the transfer of activated T lymphocytes counterbalanced the increase in these cytokines induced by the oral administration of parasite extract alone, as we found intermediate levels of IL‐10 and TGF‐β in the T cell and the AgTc groups (Fig. 5A and B). IL‐12 was not observed in the liver of any experimental group (data not shown). In the plasma of mice that received activated T cells, we observed an increase in the proinflammatory cytokines TNF, IL‐6, and MCP‐1, with no positive results for the production of IL‐12, IFN‐γ, IL‐10, or TGF‐β in any of the groups (data not shown). One pathway that could be involved in the modulation of the hepatic environment after the transfer of activated peripheral T lymphocytes relies on the metabolic enzyme indoleamine 2,3‐dioxygenase 1 (IDO1). This enzyme is expressed by cells such as macrophages and DCs and converts the essential amino acid tryptophan (Trp) into downstream catabolites known as kynurenines (Kyn), leading to T lymphocyte functional down‐regulation. However, we observed no reduced expression of IDO‐1 in the T cell and/or T cell+AgTc groups compared with that of the Control group (Supplemental Fig. 2A–C), suggesting that IDO‐1 does not play a role in the more proinflammatory status observed in the liver after the T cell transfer. Moreover, we observed an apparent up‐regulation of IDO‐1 in the AgTc group (Supplemental Fig. 2D) and the expression of IDO‐1 was mostly restricted to zone 1 of the hepatic lobules.

### 3.3 Proinflammatory function of activated liver T lymphocytes

Our results suggest that activated peripheral T lymphocytes can lead to a hepatic inflammatory response, which could reduce the hepatic tolerance to orally administered *T. cruzi* PAMPs. We then repeated the experiments and evaluated whether hepatic damage followed the oral challenge with parasite extract in recipient mice. We observed no increase in hepatic damage in mice that received PAMPs only by gavage (AgTc group) when compared with that of the Control group (Supplemental Fig. 2E). However, after T cell transfer and oral challenge, we observed an increase in hepatic damage of approximately 8‐fold in the T cell+AgTc group and of approximately 4‐fold in the T cell group (Supplemental Fig. 2E).

**FIGURE 6:**
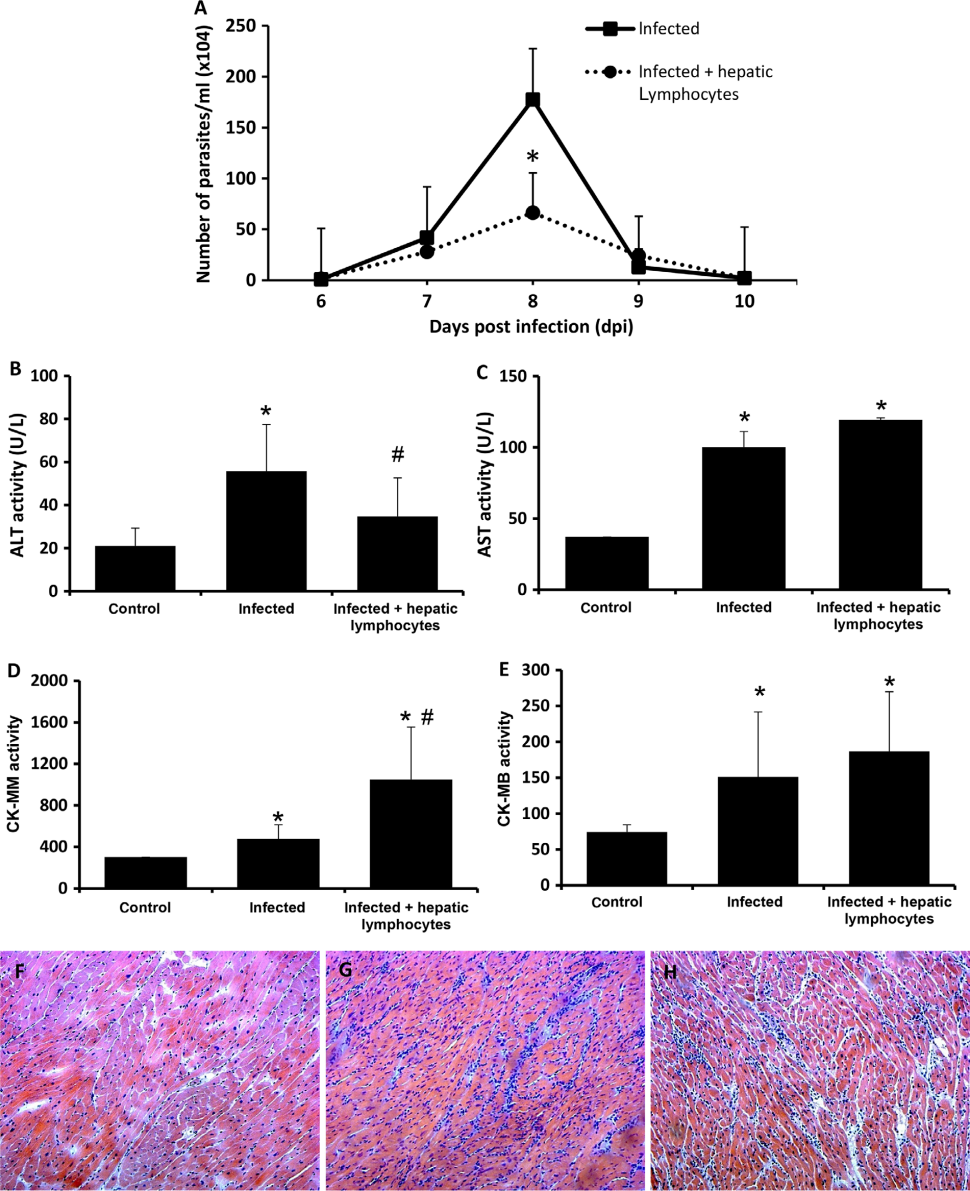
**Activated intrahepatic T lymphocytes in *T. cruzi* infection**. Thirteen‐week‐old male GFP mice were i.p. treated with *T. cruzi* extract, and activated splenic T lymphocytes (CD3^+^CD44^high^CD62‐L^−^CD197^−^) were purified. Then, 5 × 10^4^ activated GFP^+^ spleen cells were transferred to syngeneic C57BL/6 recipient mice that concomitantly received parasite extract by gavage (equivalent to 1 × 10^7^ parasites). Fifteen days after the transfer, the activated GFP^−^ intrahepatic T cells from the recipients were purified, and 1 × 10^6^ cells were transferred to C57BL/6 mice immediately before in vivo infection using 1 × 10^4^ trypomastigote forms of *T. cruzi* (infected + hepatic lymphocytes group). The other groups received 1 i.p. injection of PBS (Control) or were only infected (infected group). Parasitemia was evaluated in blood on the indicated days postinfection (dpi) (**A**) and ALT (**B**), AST (**C**), CK‐MM (**D**), and CK‐MB (**E**) were evaluated in plasma 15 days after infection. H&E staining was performed in cardiac slices from Control (**F**), infected (**G**), and infected + hepatic lymphocytes (**H**). One experiment was performed with 5–6 mice per group. The results were analyzed using ANOVA and Tukey's as the post hoc test and * indicates *P* < 0.05 compared with the control group; # indicates *P* < 0.05 comparing infected plus hepatic lymphocytes with the infected group

As we observed that the adoptive transfer of activated peripheral T lymphocytes induces the activation of αβ IHLs, we decided to evaluate the function of these activated hepatic T cells after in vivo infection with *T. cruzi*. We repeated the experiments of adoptive transfer of activated peripheral T cells using GFP donor mice and performed a second sorting in which downstream activated GFP^−^ intrahepatic T lymphocytes were purified and transferred to mice that were immediately infected with *T. cruzi*. We observed that the transfer of activated hepatic T lymphocytes reduced circulating parasitemia at 8 dpi (Fig. 6A) and hepatic damage, as ascertained by blood levels of ALT (Fig. 6B) when compared with those of infected mice. There were increased levels of blood AST in both groups of infected mice, regardless of the transfer of activated IHLs (Fig. 6C). As AST levels are affected by skeletal muscle damage, and previously published data indicated that *T. cruzi* infection occurs in muscle tissues,^17^ we evaluated the levels of CK‐MM as an indicator of skeletal muscle damage and observed higher levels in both groups of mice after infection (Fig. 6D). Our AST and CK‐MM data suggest that skeletal muscle damage is inflicted by IHLs after infection. Moreover, we found no changes in the levels of circulating CK‐MB, which is an indicator of cardiac damage, in the infected groups (Fig. 6E), and there were no differences in cardiac inflammatory infiltration in either group of infected mice (Fig. 6G and H). As expected, we did not observe inflammatory infiltration in control mice (Fig. 6F). Our results suggest that activated hepatic T lymphocytes can modulate *T. cruzi* infection in vivo, although there was no effect on *T. cruzi*‐induced myocarditis.

## 4 DISCUSSION

Although the liver is the only nonsecondary lymphoid tissue that sustains full activation of CD4^+18^ and CD8^+^ T cells,^19^ the immune response within the organ follows no conventional rules for T lymphocyte physiology. The hepatic environment turns T lymphocytes priming into a tricky function for several reasons. In contrast to the periphery, hepatic APCs, such as KC and dendritic cells, express low levels of costimulatory molecules and MHC‐peptide complexes and produce higher levels of IL‐10, TGF‐β, and PGE_2_. Sentinel LSECs express inhibitory molecules, such as B7‐H1, LSECtin,^20^ and CD95L (Fas‐L). In the liver, such characteristics usually lead to T lymphocyte anergy, apoptosis, and hyporesponsiveness, induction of Treg cells, activation‐induced cell death, inhibition of cytotoxic T lymphocyte function, and other effects.^21^ However, it has been shown that after viral infection, hepatocarcinoma, and other conditions, the hepatic environment shifts and favors antigen‐dependent immunity.^21^ Considering the multitude of gut‐derived PAMPs percolating the organ and the PAMPs of pathogens after eventual infections it is not clear how the liver discriminates which ones should be recognized as danger signals for an effector immunologic response. Using *T. cruzi*‐derived PAMPs orally administered, we observed that the liver has a limited capacity to discriminate danger from nondanger molecules and remains with characteristics of immunologic tolerance. We then propose that activated peripheral T lymphocyte, which recognized danger molecules in secondary lymphoid organs, are mainly responsible for the shift of intrahepatic cells towards a proinflammatory immune response in the liver. A cooperation of the peripheral immune response on the hepatic environment. This proposed mechanism addresses a not yet proven aspect of the hepatic immune response that may not be limited to the infection.

It seems that to a certain point, the default hepatic immune response to oral PAMPs is immune tolerance. This is illustrated by the orally administered *T. cruzi* PAMPs that reached the liver through the portal vein (OralAgTc and AgTc groups). In this case, the antigens would first interact with MALT, which are usually associated with immune tolerance, and then reach the already tolerogenic hepatic stroma. We believe that the migration of activated peripheral T cells to the liver and their effect over the hepatic ambient is key to the response profile that is locally triggered.

We observed that a single oral treatment with *T. cruzi* extract (oral AgTc group), which was a set of PAMPs that had never been encountered by the hepatic immune cells, induced no proinflammatory pathways in the liver and actually increased stromal IL‐10 and B7‐H1. When the mice received the parasite extract orally and received an (oral) challenge after 15 days (AgTc group), the tolerogenic nature of the liver became more evident, with increased B7‐H1, CTLA‐4, IL‐10, and TGF‐β. However, single i.p. injection of parasite extract (i.p. AgTc group) led to a very different scenario in the liver, with increased effector/T_em_ IHLs (CD44^high^CD127^−/+^CD62L^−^CD197^−^), PD1 down‐regulation (data not shown), and increased IL‐10, TNF, and IL‐6. The same pool and amount of antigen extract with different routes of antigen administration elicited different responses.

Taken together, our data suggest that pathways for danger discrimination in the liver are less effective within the organ itself. In fact, in the literature, it has been extensively described that antigen‐dependent immunity can be established in the liver, but the switch to subvert the default tolerant nature has not been unequivocally described, as the mere presence of PAMPs in the organ did not fully explain this shift. It was proposed before that “an appropriate stimulation” for hepatic immunity consists of the introduction of elevated quantities and/or constant exposure to PAMPs, live bacteria or viral infection,^22^ which must be sensed mostly by TLRs and inflammasomes.^23^ Regarding the first point, the levels of PAMPs from commensal microbiota products in the liver are already very high, being approximately 100‐fold higher between portal blood and peripheral venous blood for LPS, a representative molecule for microbiota products.^24^ Therefore, it is unlikely that the amount of PAMPS is the key to triggering hepatic immunity, and our proposal based on the relevance of peripheral T lymphocytes in the hepatic environment provides additional nonconflicting explanations for liver immunity. In the case of LPS‐induced acute liver damage, for example, systemic administration of heat‐killed *Propionibacterium acnes* rendered mice highly susceptible to LPS.^25^ The 50% lethal dose (LD50) of LPS in nontreated naïve mice is approximately 50 mg/kg, whereas in acnes‐primed mice, it is less than 50 µg/kg. The authors determined that surface TLRs, IL‐12,^26^ IL‐18, and KCs are important for this liver sensitization. However, in light of our present results, it is possible that activated peripheral T lymphocytes induced by previous antigen exposure are responsible for the shift to the proinflammatory and responsive environment against LPS challenge in the liver. Indeed, the hepatic environment is already exposed to high levels of LPS, but previous peripheral sensitization is part of the induction of LPS‐dependent liver damage. A similar model of mice infected with *Salmonella typhimurium* rendered CD4^+^ T cells responsive to a subsequent LPS injection by producing IFN‐γ in the liver.^27^

The constant exposure of the hepatic environment to gut‐derived antigens also does not explain how the liver discerns danger PAMPs from gut‐derived PAMPs. However, this may at least partially explain the constant tolerance to LPS and other intestinal PAMPs in the liver.^28^ Finally, even the presence of live bacteria in the liver does not necessarily lead to inflammation and immunity in the organ. Under normal conditions, small amounts of live bacteria escape the gut‐mucosal barrier and enter the liver. However, this liver‐restricted exposure is silent mainly due to KCs activity and intracellular destruction of bacteria with no T cell activation.^29^ However, a robust hepatic immune response is established in the case of systemic infection with bacteria such as *Ehrlichia*, an obligate intracellular LPS‐deficient bacteria and a common cause of acute liver injury.^30^

Other authors suggested that peripheral T lymphocytes could play a role in hepatic immune responses, and the hypotheses include T lymphocyte competition for antigen presentation in hepatic or peripheral lymphoid tissues,^10^ late T cell activation in the liver after peripheral antigen priming,^11^ peripheral IL‐12‐dependent reversion of liver‐induced immune tolerance,^12^ and others.^13^, ^14^ The experimental models include AIH induced in transgenic mice and expression of hepatitis viral antigens; although we believe that part of these results further supports our model, experimental limitations impaired the unequivocal interpretation of those previous data. One central point in these and other papers is that the expression of antigens is not restricted to the hepatic environment or the periphery, and this mixed antigen site presentation subverts the evaluation of the primary site of danger recognition. Intravenous injection of plasmids containing viral genes led to increased levels of transgene expression in the liver, but significant levels were also observed in the kidneys, spleen, heart, and lungs.^31^ Moreover, even with preferential expression of the transgenes in the liver, transgene‐derived viral proteins were observed in the blood up to 7 days after transfection.^32^ To avoid this experimental limitation, we adoptively transferred peripheral (splenic) T lymphocytes that had already been activated and found that a proinflammatory hepatic response to danger occurred only in the presence of these adoptive cells. In this case, part of the importance of activated peripheral T lymphocytes retained in the liver would be the induction of hepatic T cell expansion and activation. We believe that our approach significantly contributes to clarifying this central issue.

We were very interested in the IHL population of effector and T_em_ lymphocytes that were found in the liver after activated splenic T lymphocyte transfer. Therefore, we adoptively transferred these cells to recipient mice at the same time as in vivo infection and observed no modulations in cardiac damage or myocarditis, as progressive cardiac involvement is the leading cause of death in chronic symptomatic patients.^33^ However, we found reduced circulating parasitemia and increased skeletal muscle damage, suggesting that transferred activated IHLs may play inflammatory roles outside the liver, although we still do not know if, under physiologic conditions, these cells emigrate from the organ. Several published results suggest that hepatic T lymphocytes may exit the liver and play regulatory roles in the periphery, which is the other side of the coin presented here. The mechanisms of liver‐induced systemic immune tolerance are not well understood, but it has long been observed that pre‐exposure of recipient animals to donor cells through the portal vein increased their acceptance of solid tissue allografts.^34^ Moreover, pre‐exposure of soluble antigens via the portal vein leads to systemic immune tolerance.^35^ However, we are only beginning to elucidate the interplay and cross‐talk between hepatic and peripheral T lymphocytes, leading to immunity or tolerance, with an enormous potential impact on therapeutic strategies for systemic and liver pathologies.

## DISCLOSURE

The authors declare no conflicts of interest.

## Supporting information

Supporting Information

Supporting Information
