# Supplementary material for: In the presence of Trypanosoma cruzi antigens, activated peripheral T lymphocytes retained in the liver induce a proinflammatory phenotypic and functional shift in intrahepatic T lymphocyte
Source: J Leukoc Biol. 2020 Mar 23;107(4):695–706. doi: 10.1002/JLB.3A0220-399RR (PMC7383480; doi:10.1002/JLB.3A0220-399RR)
Supplement: Supplementary file 1 — Supporting Information [file JLB-107-695-s001.pptx]

## Slide 1
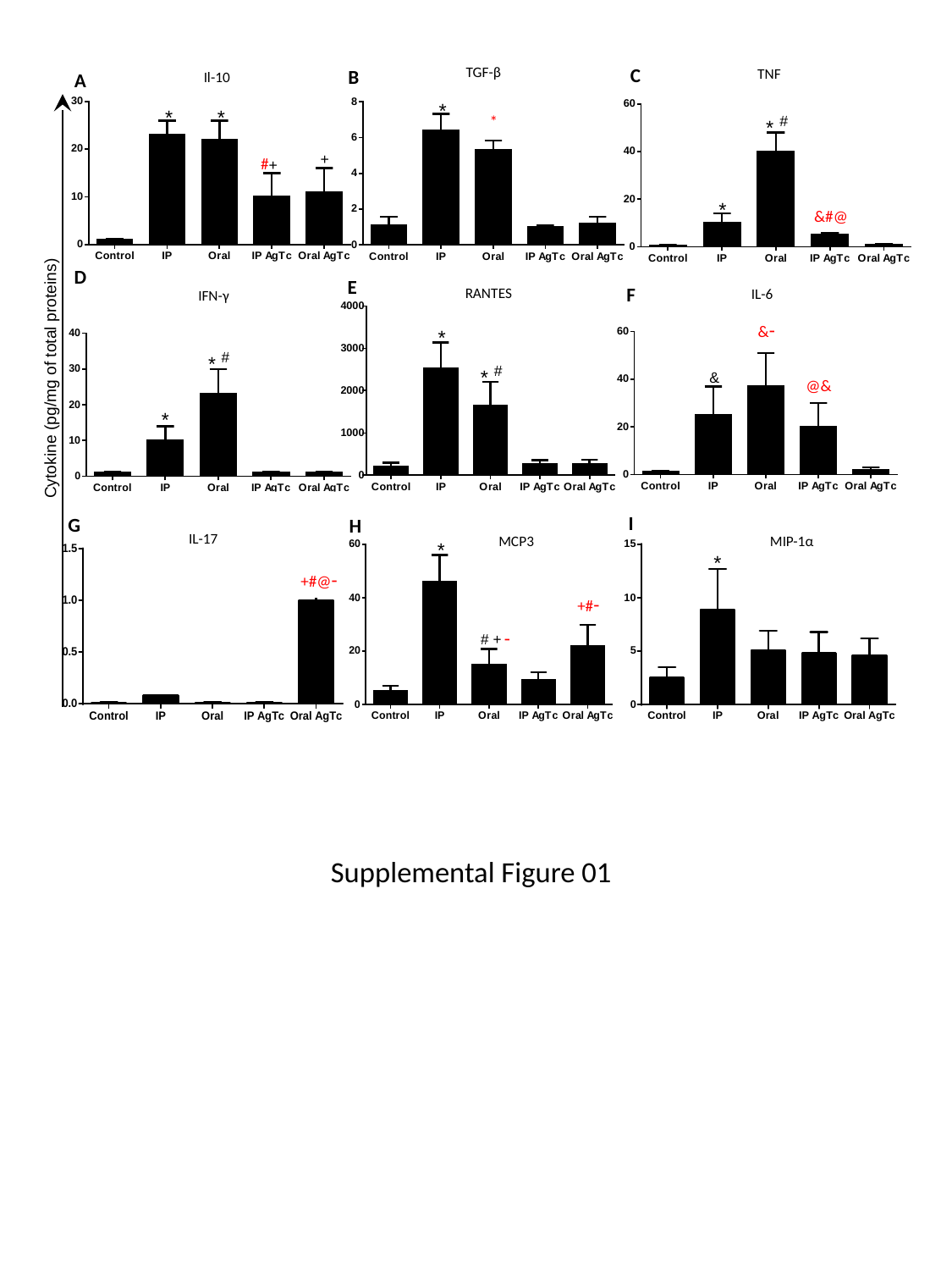

TGF-β
C
TNF
B
Il-10
A
*
#
&#@
D
E
F
RANTES
IL-6
IFN-γ
&-
Cytokine (pg/mg of total proteins)
@&
I
G
H
IL-17
MCP3
MIP-1α
+#@-
+#-
-
Supplemental Figure 01

## Slide 2
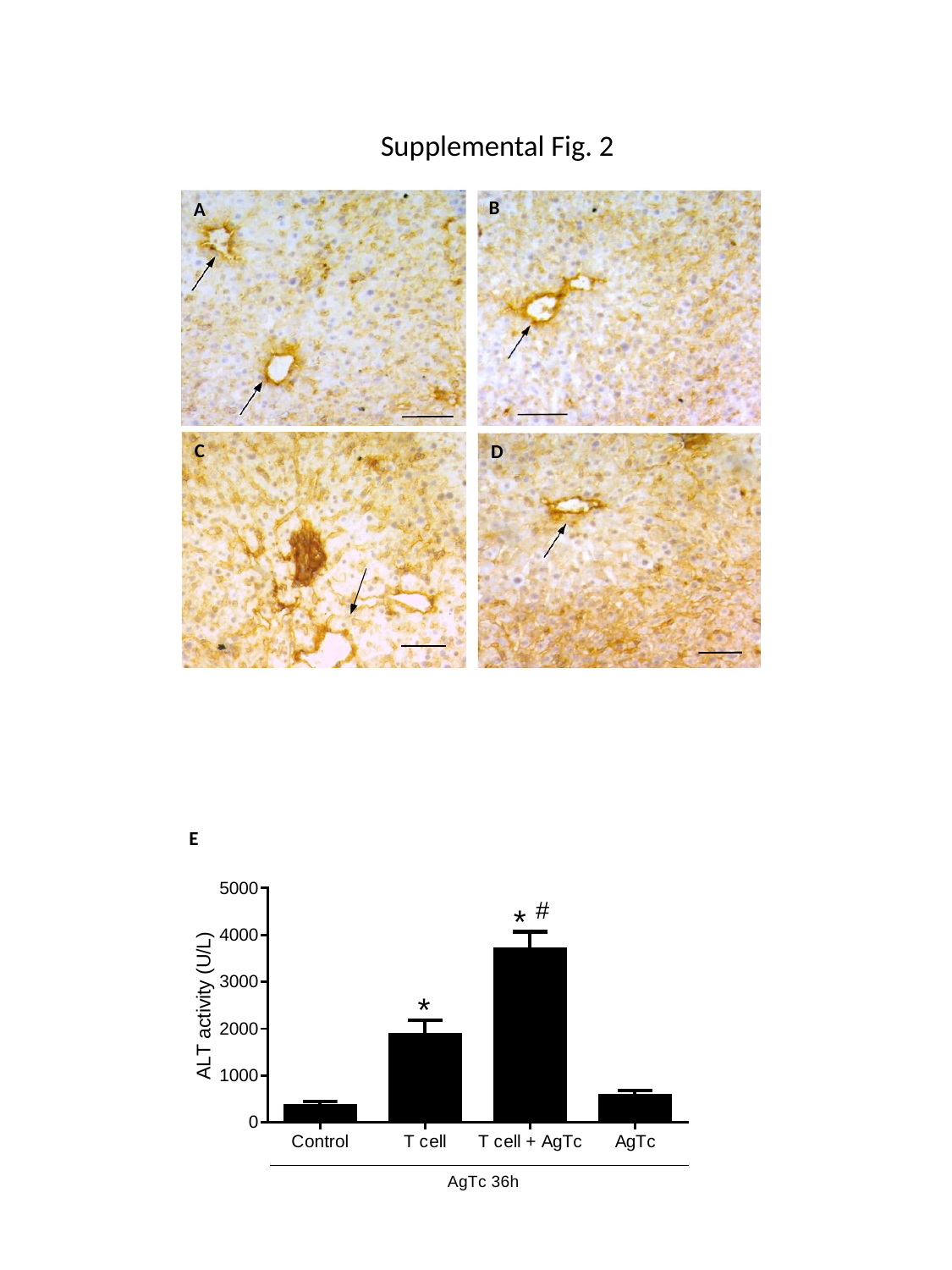

Supplemental Fig. 2
B
A
C
D
E
