# Supplementary material for: In the presence of Trypanosoma cruzi antigens, activated peripheral T lymphocytes retained in the liver induce a proinflammatory phenotypic and functional shift in intrahepatic T lymphocyte
Source: J Leukoc Biol. 2020 Mar 23;107(4):695–706. doi: 10.1002/JLB.3A0220-399RR (PMC7383480; doi:10.1002/JLB.3A0220-399RR)
Supplement: Supplementary file 2 — Supporting Information [file JLB-107-695-s002.doc]

**Supplemental Figure 1: Expression of cytokines in the hepatic tissue**. Thirteen-week-old male C57BL/10 mice were grouped as Control, infected (Oral or IP), and mice that received *T. cruzi* extract intraperitoneally (IPAgTc) or by gavage (OralAgTc). After 15 days, the livers were perfused and macerated, and the production of IL-10 (A), TNF (C), IFN-γ (D), RANTES (E), IL-6 (F), IL-17 (G), MCP-3 (H), and MIP-1α (I) was determined by flow cytometry. The production of TGF-β (B) was evaluated by ELISA. Data represent the mean and standard deviation of three independent experiments with five or six mice per group. The concentration of cytokines was normalized and is expressed in pg per mg of total proteins in tissue extract. The results were analyzed using Kruskal-Wallis and Dunn’s as the post hoc test and * indicates P <0.05 compared to the Control, IP AgTc and Oral AgTc groups; # indicates P <0.05 compared to the IP group; + indicates P <0.05 compared to the Control group; - indicates P <0.05 compared to the IP AgTc group; & indicates P <0.05 compared to the Control and Oral AgTc groups; @ indicates P <0.05 compared to the Oral group.

**Supplemental Figure 2: Evaluation of IDO-1 in the hepatic tissue after activated peripheral T cells transfer and hepatic damage**. Activated splenic T lymphocytes (CD3^+^CD44^high^CD62-L^-^CD197^-^) were purified from 13 week-old male C57BL/10 mice after IP treatments with *T. cruzi* extract. Recipient mice were divided as follows: “Control”, mice that received PBS by gavage (A); “T cell”, mice that received IP 5x10^4^ T cells/mouse and PBS by gavage (B); “T cell + AgTc”, mice that received IP 5x10^4^ T cells/mouse and parasite extract, the equivalent to 1x10^7^ parasites by gavage (C); and “AgTc”, mice that received PBS IP and parasite extract, the equivalent to 1x10^7^ parasites by gavage (D). 15 days after the transfer/treatment, all mice received the equivalent to 1x10^7^ parasites by gavage and were euthanized after 36 hours. IDO-1 expression was evaluated by immunohistochemistry in two individual experiments with four to five mice per group. The ALT (E) assay was based on enzyme activity in plasma and determined by spectrophotometry. Data represent mean and standard deviation of three independent experiments with five to six animals per group. * indicates P <0.05 compared to the Control and AgTc groups; # indicates P <0.05 compared to the T cell group. Arrows indicate central veins. Bar = 100µm.
